# Diagnosis of tuberculosis from smear-negative presumptive TB cases using Xpert MTB/Rif assay: a cross-sectional study from Nepal

**DOI:** 10.1186/s12879-019-4728-2

**Published:** 2019-12-30

**Authors:** Priyatam Khadka, Januka Thapaliya, Ramesh Bahadur Basnet, Gokarna Raj Ghimire, Jyoti Amatya, Basista Parsad Rijal

**Affiliations:** 1 Medical Microbiology, Tri-Chandra Multiple Campus, Kathmandu, Nepal; 2 Tribhuvan University Teaching Hospital, Kathmandu, Nepal; 3 National Tuberculosis Center (NTC), Bhaktapur, Nepal

**Keywords:** Xpert MTB/Rif assay, *Mycobacterium tuberculosis*, Line probe assay, MDR-TB, Smear-negative

## Abstract

**Background:**

In most developing countries, smear-negative pulmonary TB (SNPT) often gets missed from the diagnosis of consideration, though it accounts 30–65% of total PTB cases, due to deficient or inaccessible molecular diagnostic modalities.

**Methods:**

The cross-sectional study enrolled 360 patients with clinical-radiological suspicion of SNPT in Tribhuvan University Teaching Hospital (TUTH). The patient selection was done as per the algorithm of Nepal’s National Tuberculosis Program (NTP) for Xpert MTB/RIF testing. Participants’ demographic and clinical information were collected using a pre-tested questionnaire. The specimens were collected, processed directly for Xpert MTB/RIF test according to the manufacturer’s protocol. The same samples were stained using the Ziehl-Neelsen technique then observed microscopically. Both findings were interpreted; rifampicin-resistant, if obtained, on Xpert testing was confirmed with a Line Probe Assay.

**Result:**

Of 360 smear-negative sputum samples analyzed, 85(23.61%) found positive while 3(0.8%) of them were rifampicin resistance. The infection was higher in males, i.e. 60(25.3%) compared to female 25(20.3%). The age group, > 45(nearly 33%) with median age 42 ± 21.5, were prone to the infection. During the study period, 4.6% (515/11048) sputum samples were reported as smear-positive in TUTH. Consequently, with Xpert MTB/RIF assay, the additional case 16.5% (*n* = 85/515) from smear-negative presumptive TB cases were detected. Among the most occurring clinical presentations, cough and chest pain were positively associated with SNPT. While upper lobe infiltrates (36.4%) and pleural effusion (40.4%) were the most peculiar radiological impression noted in PTB patient. 94 multi-drug resistant(MDR) suspected cases were enrolled; of total suspects, 29(30.8%) samples were rifampicin sensitive, 1(1.06%) indeterminate, 3(3.19%) rifampicin-resistant while remaining of them were negative. 2(2.2%) MDR cases were recovered from the patient with a previous history of ATT, of total 89 previously treated cases enrolled However, a single rifampicin-resistant from the new suspects.

**Conclusion:**

With an application of the assay, the additional cases, missed with smear microscopy, could be sought and exact incidence of the diseases could be revealed.

## Background

Tuberculosis is a treatable and curable disease (if early diagnosis of etiology and its drug resistance status could be made) but has been existing as a major public-health-threat around the globe [1]. The reasons behind this unyielding infection are due to inaccessible or lacking diagnostic tool which carries higher precision and over-relying on clinical presentations, chest radiography and/or sputum smear microscopy—in most health centers of developing countries [2–4]. As an air-borne infection, TB in one hemisphere ultimately relocates to others. Billions of dollars are being spent in developed nations; however, is worthless and these nations could not skip alone from the infection. The expanses could be fructiferous only if interrupting transmission chains from developing nations could be possible. For which, developing nations should be boosted continously with the collaborative support (financial and technological support) from developed nations.

Globally, 10.4 million people get infected with TB, of which 41% were from South East Asia. Turning to Nepal, nearly half of the population is infected with tuberculosis. About Nepal’s National TB Program (NTP) report in 2015/16, 32,056 presumed cases were registered where 75% of them were bacteriologically confirmed as *Mycobacterium tuberculosis* [5]. It has been estimated, each year, approximately 166 per 100,000 population develop TB, 5000–7000 death reported, and 8000–10,000 cases are predicted of being missed [5]. In most developing countries due to deficient or inaccessible molecular diagnostic modalities, SNPT often gets missed from the diagnosis of consideration—though it accounts for 30–65% of total PTB cases [6, 7]. Hence, tracing and treating these cases could be an auxiliary for reducing the global burden.

To address this burgeoning threat, world health organization (WHO) endorsed Xpert MTB/RIF assay as an accurate, feasible, rapid, affordable, and near-point-care TB diagnostic test for resource-limited settings of developing countries in December 2010. As collaborative efforts of global fund and NTP, Nepal the test was made available, for all patients requiring the test, in absolutely free of cost. However, only limited health centers provide this facility since fund circulation in every public hospital is yet to be done, and public-private health center’s partnership is yet to be established, for making the test accessible to needful.

The study was aimed to diagnose tuberculosis from smear-negative presumptive PTB cases using Xpert MTB/RIF assay.

## Methods

### Study design and settings

A cross-sectional study was conducted among smear-negative but presumptive TB patients. The study was conducted at TUTH, the largest public hospital in Nepal, between13^th^ April 2016 -14th April 2017. This is the only hospital where patients from the whole nation (with any economic class) come for treatment due to its’ best-health-care facilities and super-specialty-care at an affordable cost. Before the introduction of Gene Xpert, the laboratory setting of this hospital was limited with AFB smear microscopy and *Mycobacterium tuberculosis* (MTB) culture but no drug susceptibility test (DST) facility. After then, with an aid of Global Fund and NTP the diagnostic test was made accessible as cost free services for all patients meeting algorithms set by NTP: presumptive PTB cases with abnormal chest X-ray, people living with HIV/AIDS (PLHA), treatment failure or previously treated cases and MDR suspects. Only those eligible patients were selected for the study.

For the estimation of actual SNPT cases in 1 year, the smear-microscopy result over the study period was extracted from the laboratory record file where 4.66% (515/11048) positive cases were recorded.

Participants’ demographic and clinical information were collected using a pre-tested questionnaire—written in the local language. The clinical evaluation and classification as MDR-TB cases were done by an expert chest physician; chest X-ray features were classified as per radiologist reports. The patient with abnormal chest X-ray (upper lobe infiltrates, pleural effusion, diffuse infiltrates, cavitary lesions, other infiltrates, consolidation, other abnormalities) and having clinical presentations: persistent cough (≥2 weeks), fever, drenching night sweats, weight loss (> 1.5 kg in a month), loss of appetite, malaise, and shortness of breath or chest pain; were presumed as PTB. However, as MDR-TB, based on the smear results, previous treatment history, and no clinical improvement in response to the ATT (anti-tubercular therapy) after 2 months.

### Inclusion and exclusion criteria

All smear-negative presumptive TB cases, either from previously treated cases or new suspects, and MDR suspects were enrolled. All positive cases diagnosed with smear microscopy were excluded, however. During our study period, 3 HIV positive patients were registered; nevertheless, not included in our study since all of them had smear-positive sputum.

### Xpert MTB/RIF assay and MDR confirmation

All the procedures for gene Xpert testing were followed as per the manufacturers’ specifications and guidelines [8]. In brief, 0.5 ml of expectorated sputum sample and Xpert sample reagent was added in the ratio 1:2 and was vortexed twice with 15 min incubation at room temperature until the sample gets emulsified completely. After then, 2 ml of the mixture was transferred to Xpert test cartridge; the cartridge was then loaded into Xpert machine. Gene Xpert DX system, interprets the results, from measured fluorescent signals and display automatically either MTB complex detected, not detected, or rifampicin-resistant (if present) only after 90mins.

Cases of rifampicin-resistance detected by Xpert were confirmed in the NTC Laboratory using MTBDR plus line probe assay (Hain Life science Germany) as per the manufacturers’ instructions. In brief, bacterial DNA presumed as Rif resistant from sputum sample was extracted using cetyl-trimethyl ammonium bromide (CTAB) method which was then amplified, purified and hybridized with strips of MDRTB plus. The evaluation and interpretation of the results were done as per the interpretation chart provided with the kit.

### Data management and analysis

The data obtained was entered in Microsoft Office Excel 2007 and analyzed by Statistical Package for Social Sciences (SPSS) version 16.0. Frequencies and percentages were calculated, and the odds ratio and nominal 95% confidence intervals (CIs) were presented for elucidating the association between variables. A two-sided *p*-value < 0.05 was considered significant for all analyses. The sample size calculation was based on the prevalence rate (241 per 100,000 population) in Nepal [4, 5]. We estimated the sample size of 281 at 95% confidence level.

## Result

### Patients’ demographics

**Fig. 1:**
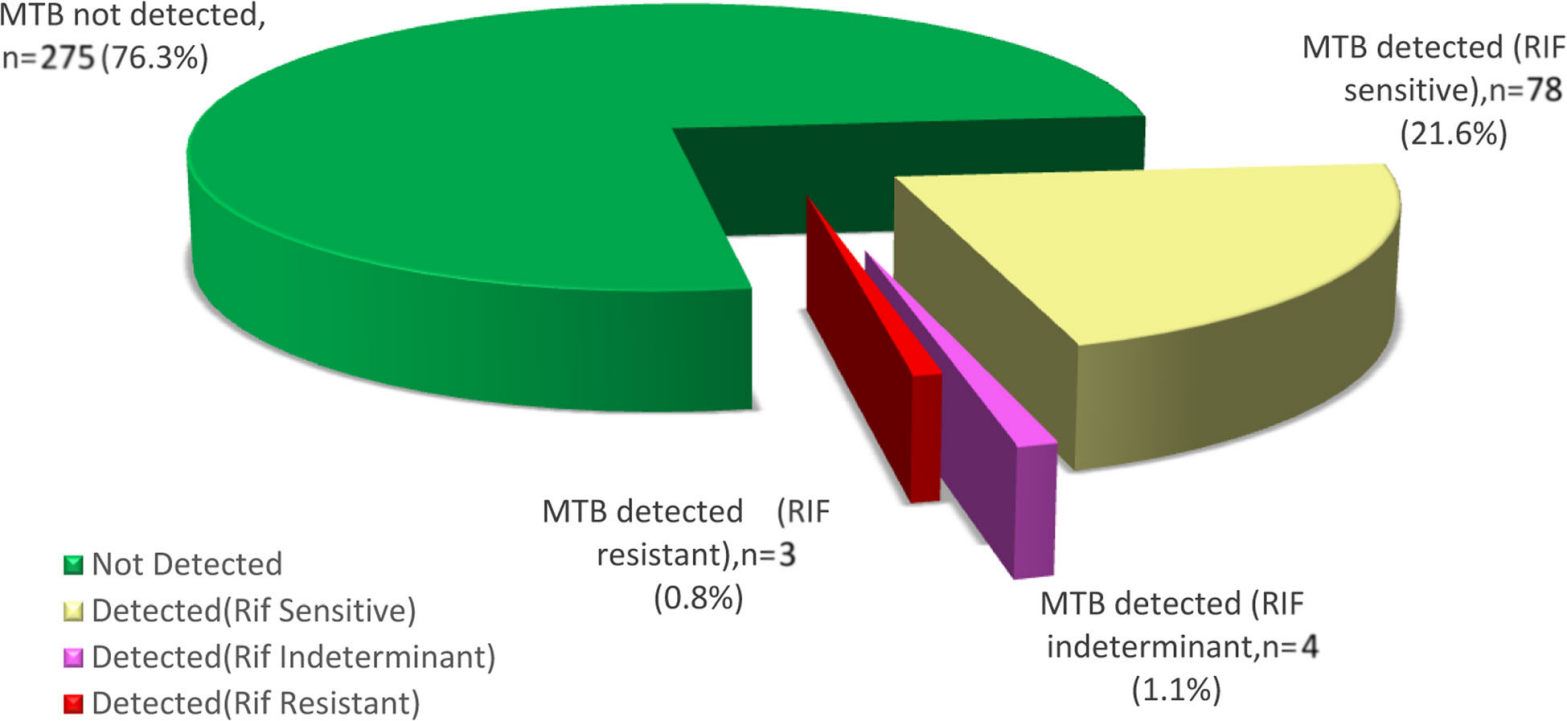
The total case detection of SNPT with Xpert MTB/RIF Assay is shown in the pie chart. Each portion of pie chart indicates the number/percentage of enrolled cases and their result

A total of 360 smear-negative sputum samples (including 237 male and 123 female clinically suspected pulmonary tuberculosis patients) were tested with Xpert MTB/RIF assay. Among enrolled smear-negative cases, 85(23.61%) were positive PTB; where 3(0.8%) rifampicin resistance cases observed (Fig.1). The infection was higher in males, i.e. 60(25.3%) compared to female 25(20.3%). The age group, > 45(nearly 33%) and below 15(20%) years, with median age 42 ± 21.5, found prone to the infection. The higher percentage of new suspects 63(23.5%) found positive on gene Xpert testing compared to previously treated cases 20(22.5%) while highest was among loss to follow up 2(66.6%) (Table 1).

**Table 1:** Patients’ demographics

Patients demographics	Smear-negative PTB not detected with Xpert MTB/RIF assay (%)	Smear-negative PTB detected with MTB/RIF assay (%)	Total	OR	95% CI	*P* value
Gender						
Male	177(74.7)	60(25.3)	237	1.33	0.78–2.5	0.29
Female	98(79.6)	25(20.3)	123			
Age group						
< 14 year	36(80)	9(20)	45	_	_	_
15 to 29 years	65(89.9)	8(10.1)	73	0.5	0.21–1.39	0.2
30 to 44 years	62(81.6)	14(18.4)	76			
45 to 59 years	53(67.9)	25(32.1)	78	0.95	0.50–1.84	0.93
> 60 years	59(66.3)	29(33.7)	88			
Clinical history						
New suspects	205(76.5)	63(23.5)	268	1.06	0.59–1.87	0.84
Previously treated	69(77.5)	20(22.5)	89			
Loss-to-follow up	1(33.4)	2(66.6)	3	_	_	_

*OR* Odds ratio, *CI* Confidence Interval

During the study period, 4.6% (515/11048) sputum samples were reported as smear-positive in TUTH. Our study sum-up the additional case of pulmonary tuberculosis i.e. 16.5% (*n* = 85/515), which were missed on the smear-microscopy.

### Clinical presentations

Among the most occurring clinical presentations, cough and chest pain were more evident in SNPT cases and are significantly associated too. However, other clinical presentations: fever, weight loss, and night sweat, found as non-specific presentations. (Table 2).

**Table 2:** Characterization of PTB cases in terms of clinical presentation

Clinical presentation		Result of gene Xpert MTB/Rif		Total	Odds ratio	95%CI	*P* value
		Not detected (%)	Detected (%)				
Fever	Y	207(74.7)	70(25.3)	277	1.5	0.82–2.85	0.17
	N	68(81.9)	15(18.1)	83			
Cough	Y	242(74.7)	82(25.3)	324	3.7	1.11–12.48	0.03
	N	33(91.7)	3(8.3)	36			
Weight loss	Y	229(76.1)	72(23.9)	301	1.1	0.57–2.17	0.75
	N	46(78.0)	13(22)	59			
Chest pain	Y	87(59.1)	60(40.9)	147	5.2	3.05–8.82	0.001
	N	188(88.2)	25(11.8)	213			
Night sweat	Y	146(76.0)	46(24)	192	1.04	0.64–1.7	0.86
	N	129(76.8)	39(23.2)	168			

### Radiological impression on chest X-ray

Of the total enrolled patient, 345 were presented with an abnormal radiological impression on chest X-ray while 15 of them had normal findings. Excluding patients with normal radiological findings, 23.38% (*n* = 83/355) with abnormal chest X-rays had acquired PTB. The upper lobe infiltrates (36.4%) and pleural effusion (40.4%) were evident in PTB patient; nevertheless, other impressions, like hilar/mediastinal lymphadenopathy (19.2%), cavitary lesion (15.6%), diffuse infiltration (12.2%), segmental/lobar consolidation (3.2%), were also noted. However, these impressions were not statically significant. (Table 3).

**Table 3:** Characterization of PTB cases in terms of clinical presentation chest X-ray features

Features on chest X-ray	Result of Xpert MTB/Rif assay		Total(*n*)
	Not detected (%)	Detected (%)	
Upper lobe infiltrate	70a(63.6)	40b(36.4)	110
Pleural effusion	34a(59.6)	23b(40.4)	57
Hilar /mediastinal lymphadenopathy	21a(80.8)	5a(19.2)	26
Cavitary lesion	27a(84.4)	5a(15.6)	32
Normal	13a(86.7)	2a(13.3)	15
Diffuse infilteration	29a(87.9)	4a(12.1)	33
Abnormal	51a(91.1)	5b(8.9)	56
Segmental/lobar consolidation	30a(96.8)	1b(3.2)	31
Total Count	275(76.4)	85(23.6)	360

*Each subscript letter denotes a subset of result of Xpert MTB /RIF test categories whose column proportions do not differ significantly from each other at the .05 level

### MDR case findings with gene Xpert and its’ confirmation

94 MDR presumptive cases were enrolled. Among them, 29(30.8%) samples were rifampicin sensitive, 1(1.06%) indeterminate, 3(3.19%) rifampicin-resistant while remaining of them were negative. 2(2.2%) MDR cases were recovered from the patient with a previous history of ATT, of total 89 previously treated cases enrolled while a single case from the new suspects.

**Fig. 2:**
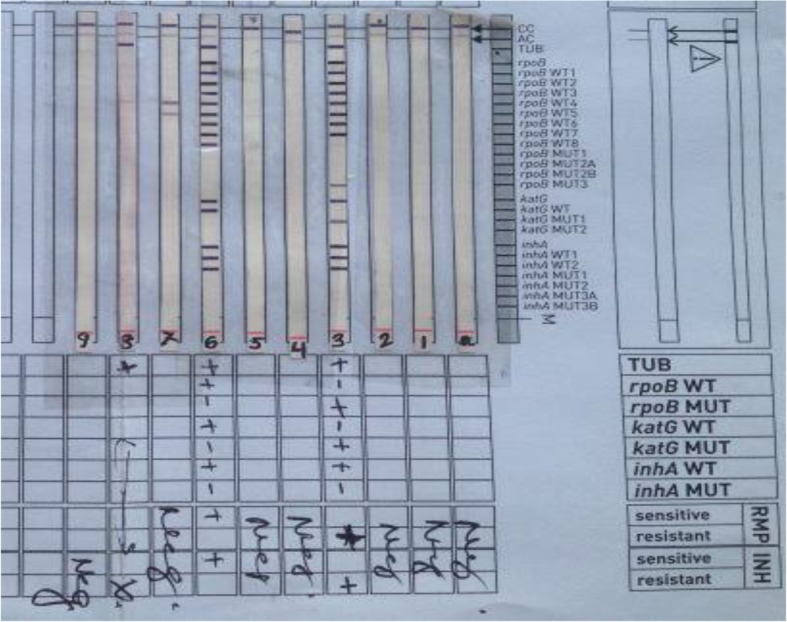
Line probe assay showing mutated genomic sequences

MDR confirmation was done with Line Probe Assay where distinct bands revealing the genomic sequences i.e. TUB(+), rpoBWT(−), rpoBMUT(+), kat GWT(−), kat GMUT(+), inhAWT(+), inhAMUT(−); RMP (resistant), INH (resistant) was observed in strip 3 (Fig. 2).

## Discussion

SNPT often gets missed from the diagnosis of consideration—particularly in developing countries like Nepal—though it accounts 30–65% of total PTB cases [7, 6]. With this backdrop, the study was conducted to discover the actual incidence of PTB among presumptive cases which was not done before in Nepal. The application of Xpert MTB/RIF assay, in our setting, substantially increases the confirmed PTB cases. During our research period, 4.6% smear-positive PTB cases reported at TUTH while upon execution of gene Xpert as a diagnostic tool, the rate surged to 16.5% (*n* = 85/515). Our study is consistent with other studies, conducted in developing countries, which has suggested the benefit of Xpert in smear-negative patients [9–12].

We aimed to include all presumptive cases, the infective forms i.e. PTB, and tries to break the transmission chain with speedy diagnosis as far as possible. Hence, we opted for the Xpert MTB/RIF assay. In most, research studies and meta-analysis performed to this date, higher specificity of the test up to 99% has been observed on sputum samples [10, 13–15]. Since, our study was not an evaluation, comparisons and implementation testing of gene Xpert assay against other diagnostic tests, limited studies found conducted in Nepal encompassing like our rationale [4].

In our study, we compare, likely occurring clinical features present in PTB patients with that gene Xpert results in SNPT cases. The only clinical feature to show a statistically significant difference between the groups was cough and chest pain. As portrayed with the latest meta-analysis and perspectives study where the sensitivity of cough as a positive predictive PTB ranges from 79.9 to 82% [16, 17]. Hence, these clinical features could be one of the triaging features in optimizing the test where gene Xpert was not accessible to anyone—particularly in low-income-countries.

The cost expense of the test is another tethering truth with which the developing countries are being suffering. In most developing countries, the limited public hospital (covered with Global Fund) provides gene Xpert testing facility owing to expensive test cartridge and running cost which is nearly impossible for private health centers to afford. The test accessibility to every patient is the fundamental right and should be guaranteed by every nation. Therefore, government policy with a public-private partnership for the infection eradication is obligatory.

Ironically, in Nepal as NTP, with a policy to economize cost per cartridge on par to patient’s number and also to make accessible on targeted population, had endorsed a guideline for the test. Patient with abnormal chest X-ray, presumed MDR patient, people living with HIV/AIDS (PLHA) and those with treatment failure cases were eligible for the test as described in the method section. Relying upon the guideline, we included the chest X-ray as a triaging test. In our study, common radiological findings evident in positive cases were upper lobe infiltrates (36.4%) and pleural effusion (40.4%). A similar study was conducted in our nearby hospital and neighboring country (India) where upper-lobe infiltrates and cavitary lesions were the common features [4, 18]. Further, as to extrapolate the role of X-ray in PTB diagnosis, we compared the relationship between abnormal impressions vs. positive cases. We found, 23.38% of patients with abnormal chest X-ray had acquired PTB while Dutta et al. reported 34.4% in patients with similar features [18].

Although, higher percentage of new suspects 63(23.5%) found positive on Gene Xpert testing compared to previously treated cases 20(22.5%); 2(2.2%) MDR cases were recovered from the patient with a previous history of ATT, of total 89 previously treated cases enrolled. However, a small population of previously-treated case was included in our study, the findings portrayed similar view of resistance-trend as surveyed in 1992–1993 in the Western Cape Province where 8.6% acquired and 3.2% initial drug resistance was noted in among these patients [19]. In another studies, the highest, in range from 15 to 27.7%, MDR-TB was found to be associated in previously treated cases, however [20, 21]. Furthermore, the previously treated patients may be at high risk of extensively drug-resistant (XDR) TB too [19]. Hence, the clinicians must be cautious in treating the previously treated cases.

Of total positive cases, 3.5% (*n* = 3) were valid rifampicin-resistant (confirmed with Line probe assay); no false-positive rifampicin resistance was noted as reported in different literature with gene Xpert testing [15] [13, 22, 23]. Round the globe, about one-third of the countries had surveyed on the incidence of MDR-TB which was in between 2 and 14% [24–26]; our findings coincide within this range.

For improving patient care and abbreviating the disease transmission chain, speedy detection of tuberculosis and its drug-resistance with precision is crucial. In this perspective, endorsing gene Xpert as a diagnostic tool by WHO is the commendable action in curbing the global threat, to some extent. Further, a long-distance yet to be traveled: making it more accessible, affordable, and upgrading sensitivity of Xpert MTB/RIF assay also covering disseminated TB. Only then billions of dollars expended could be a wise investment.

### Limitations

Inability to include large samples was the major drawback of our study. Additional SNPT cases could be still missing from our study frame since the test is not of absolute accuracy. Also, we could not run the phenotypic DST and Line Probe assay for all Xpert MTB/RIF positive specimens. If it was possible, clear MDR status could be traced which might be missed even from Xpert MTB/RIF assay.

## Conclusion

For improving patient care and abbreviating the disease transmission chain, speedy detection of tuberculosis and its drug-resistance with precision is crucial. For which, gene Xpert MTB/RIF assay would be a proper option. With an application of the assay, the additional cases, missed with smear microscopy, could be sought and exact incidence of the diseases could be revealed. Further, the implementation of the test, in every developing country, if possible, could be a wise investment in restricting the global burden, as targeted by WHO.

## Data Availability

Data generated or analyzed during this study are included in this manuscript and remaining are available from the corresponding author on reasonable request.

## Abbreviations

ATT: Anti-tubercular therapy
MDR-TB: Multi-drug resistant tuberculosis
NTC: National tuberculosis center
NTP: National tuberculosis program
PLHA: People living with HIV/AIDS
SNPT: Smear-negative pulmonary TB
TUTH: Tribhuvan university teaching hospital
